# High-Performance PCR for Alleles Discrimination of Chromo-Helicase-DNA Binding Protein (CHD1) Gene in Bird Sexing

**DOI:** 10.3390/biology12020300

**Published:** 2023-02-14

**Authors:** Marcello Tagliavia, Valentina Catania, Giacomo Dell’Omo, Bruno Massa

**Affiliations:** 1 Institute for Biomedical Research and Innovation-National Research Council (IRIB-CNR), Via Ugo La Malfa 153, 90146 Palermo, Italy; 2 Department of Biological, Chemical and Pharmaceutical Sciences and Technologies (STEBICEF), University of Palermo, Viale delle Scienze, bd. 16, 90128 Palermo, Italy; 3 Department of Earth and Marine Sciences (DiSTeM), University of Palermo, Viale delle Scienze, bd. 16, 90128 Palermo, Italy; 4 Ornis Italica, Piazza Crati 15, 00199 Roma, Italy; 5 Department of Agriculture, Food and Forest Sciences (SAAF), University of Palermo, Viale delle Scienze, bd. 13, 90128 Palermo, Italy

**Keywords:** high-performance PCR, loop-mediated isothermal amplification (LAMP), fast DNA extraction, rapid bird sexing, Procellariiformes

## Abstract

**Simple Summary:**

Rapid results in genetic tests are often required, including in outdoor analyses. To date, the only fast technique is LAMP (loop-mediated isothermal amplification), which is overall less flexible than PCR. We have developed a PCR test in which very high DNA yields are achieved, allowing for direct in-tube detection of the products by fluorescence. We successfully tested this technique in rapid sexing of Procellariiformes as proof of concept, which paves the way for further development of the method to many applications.

**Abstract:**

Genetic analyses aiming at assessing the presence of specific sequences or alleles are often carried out by PCR. Sexing of most birds is nowadays based on PCR with “universal” primers and relies on the assessment of the presence of the sex-linked CHD1-Z and -W alleles. The entire workflow is relatively time-consuming, especially for batch analyses, whereas methods that allow carrying out the entire procedure in a short time are highly desirable. The only method for outdoor analyses reported so far relies on LAMP; however; it fails to work properly in Procellariiformes. Besides improving the LAMP test; we have developed a PCR-based DNA amplification procedure (named high-performance PCR); whose unique features allow it to outperform standard PCR; making possible the direct, in-tube visual reading of results. We tested it with specifically designed Procellariiformes-targeted primer sets for rapid sexing of the birds using fluorimetric detection. The protocol, combined with rapid DNA extraction, allows for fast reading of results without electrophoresis within less than 1 h from sampling. The technique could be extended to other species, as well as to many other applications.

## 1. Introduction

The identification of birds’ sex is an important tool in fields ranging from wildlife management to breeding programs, as well as livestock production. However, morphological sex assessment may be unreliable in monomorphic species, as well as in many young birds. In such cases, molecular sexing has replaced previous methods, including invasive ones such as endoscopic examination, and extended the repertoire of matrices suitable for sex assessment (e.g., feces, feathers, buccal swabs, etc.). The standard method of molecular sexing in birds relies on PCR amplification of non-coding sequences (introns) located in the sex-chromosome-specific gene encoding the Chromo-Helicase-DNA binding protein (CHD1), and mapping on the sexual W and Z chromosomes (CHD-W and CHD-Z, respectively [1]. The length polymorphisms between CHD-W and CHD-Z alleles allows for sex discrimination. The molecular sexing analysis leads to the simultaneous amplification of the two alleles in females (the heterogametic sex, one copy of Z and W), resulting in two distinct amplicons (whose length difference, across different bird species, ranges from tens to hundreds of base pairs), whereas a single band is observed in males (the homogametic sex two copies of Z chromosome) [1,2]. Alternatively, in the case of species with no length polymorphism, the procedure may include the digestion of amplicons with selected restriction endonucleases to achieve sex discrimination after gel electrophoresis [3,4]. Although the PCR technology is considered the method of choice for sex discrimination, there are some limitations that may lead to sex misidentification [5]. The same may occur due to polymorphisms in CHD-W primer recognition sites that may result in allelic drop-out (as it occurs in the Mediterranean Storm Petrel, *Hydrobates pelagicus* (Tagliavia and Massa, unpublished data), and in Eurasian woodcock *Scolopax rusticola* [6]. Moreover, PCR is rather sensitive to DNA degradation, which may result in analysis failure, especially if long fragments are to be amplified. Another limitation is due to the entire workflow (from DNA extraction using commercial kits to gel visualization after electrophoresis), which requires relatively expensive laboratory equipment for genetic analyses. Moreover, the need of performing the procedure in the laboratory (mainly due to the electrophoresis and gel visualization steps) strongly limits the possibility of outdoor analyses, which are so far reliable only using loop-mediated isothermal amplification (LAMP) [7,8].

LAMP is a DNA amplification technique that uses moderately thermophilic DNA polymerases, such as Bst (from *Bacillus stearothermophilus*) and Bsm (from *Bacillus smithii*), characterized by high processivity and strong strand displacement activity [9]. Thus, the reaction can proceed at constant temperature, with extremely high sensitivity and yields in a short time, down to 15 min [10]. The LAMP-based method allows for an easy and fast sex determination, using only a thermo-block (or a thermal cycler, if available), and does not require electrophoresis. A common limit of the LAMP analysis consists in the requirement of at least six target sequences, which condition cannot be achieved for all sequences of interest. Moreover, the highest reaction speed, which is probably one of the most attractive features of LAMP, is limited to those settings where loop primers (an additional primers pair) can be used; unfortunately, their design may be strongly limited by the target length and/or the sequence features.

Recently, the use of LAMP has been reported for outdoor molecular sexing of birds [7,11,12]. However, such reports describe a technique where a long reaction time (up to 90 min) is needed, comparable with those of PCR amplification. Moreover, the authors reported the failure of amplification in the control reaction in Procellariiformes [7].

Considering these limits of LAMP, PCR remains quite attractive due to its higher flexibility because only two short target regions are required for primer annealing, and the range of amplicon size is wider. In particular, the possibility of using primers that amplify very short regions helps to analyze samples containing degraded DNA, where a high rate of PCR failure or allelic drop-out may heavily hamper sex assessment when amplifying longer regions using some standard primers.

Unlike LAMP, however, the in-tube visual detection of PCR products is not usually considered reliable because of the significantly lower DNA yield compared with LAMP. In fact, the fluorimetric and colorimetric visual detection in the latter relies on the very high DNA concentration (and eventually related by-products such as pyrophosphate) reached in the reaction tube.

Despite such intrinsic features, PCR performances could be optimized to yield, in a short time, DNA amounts high enough to allow the visual detection of results in the reaction tubes, thus overcoming the main limitation to the use of the technique outdoors.

The efficiency of a PCR reaction is defined as the fraction of target molecules replicated in one PCR cycle [13,14]. In a properly designed PCR assay, under non-challenging conditions (such as without the interference of PCR inhibitors), target DNA amplification is expected to be achieved with a 99% efficiency [15]. However, because in general the reaction performance is much lower, several strategies have been developed to improve efficiency. The best one which proved to result in enhanced processivity and improved PCR efficiency is the use of DNA polymerases that exhibit higher affinity toward the template. Most of such commercially available enzymes are chimeric proteins, where DNA polymerase is fused to the dsDNA binding protein Sso7d from *Sulfolobus solfataricus* [16]. Sso7d is a small protein (7 kD) which binds dsDNA without any preference for specific sequences; it binds directly to dsDNA with an affinity strong enough to stabilize the DNA polymerase:DNA complex, and weakly enough as to enable the fusion protein to slide along the DNA template during nucleotides incorporation. Moreover, such fusion has been demonstrated to improve processivity without affecting the catalytic activity or thermal stability of the enzyme.

Taq and Pfu DNA polymerases have been shown to be five times faster in amplifying targets when associated with Sso7d fusion, compared to the native enzymes, which reduces the extension time during PCR amplification reaction [16]. Moreover, Sso7d fusion increases tolerance to higher concentrations of salts and PCR inhibitors (such as heparin, humic acids, tannic acid, cellulose, and trypsin), making less challenging the use of crude and/or complex matrices such as blood or soil as templates. Furthermore, the higher processivity and DNA affinity, together with the salt requirement, allow very short annealing time, thus contributing to shorten the reaction times.

We aimed to use such enzymes to set reactions in which DNA yields high enough to be detected by fluorescence by the naked eye could be achieved. We set up a new PCR-based analysis named “high-performance PCR”. This approach shares many features with LAMP, including the short reaction time (compared to standard PCR) and the possibility of reading results visually (in-tube). Moreover, it has the potential to speed-up the PCR analysis of batch of samples in the laboratory, and to be employed for outdoor analyses, as well.

The aim of the work was to improve the overall workflow for alleles detection in molecular sexing, combining a fast and reliable DNA extraction with improved amplification and results visualization.

The latter was achieved through the optimization of high-performance PCR. It was applied, as a proof-of-concept, to molecular sexing of Mediterranean Procellariiformes, using specifically designed primers, based on newly sequenced regions. This is, to our knowledge, the first report of the development of such a technique that we applied to sexing of these pelagic birds in this study. Noteworthy, the new PCR primers proved to be effective even on samples in which standard PCR primers failed to amplify.

Moreover, novel primers set for sexing of the same birds by LAMP is proposed, as an alternative to the existing ones.

A further workflow implementation was achieved through a robust and extremely simple DNA extraction procedure based on an alkaline treatment that ensures fast lysis and the inactivation of PCR inhibitors [17,18,19] that, combined with both amplification methods, allow to get results within less than one hour.

In both protocols, the high primers’ specificity and performances, together with highly processive DNA polymerases, allow for robust, sharp, and unequivocal results.

## 2. Materials and Methods

### 2.1. Samples and DNA Extraction

Blood samples from wild birds, 30 males and 50 females of Scopoli’s Shearwater (*Calonectris diomedea*), 20 males and 25 females of European Storm Petrel (*Hydrobates pelagicus melitensis*), 3 males and 3 females of Yelkouan Shearwater (*Puffinus yelkouan*), previously sexed by PCR with standard primers (CHD2550 and CHD2718) have been employed to test and validate the method. Further *C. diomedea* samples were employed (1200 of blood and 130 of feathers), among which males and females resulted nearly equally represented.

Samples were collected during the breeding seasons. Freshly sampled blood (one single drop of 1–3 μL or, alternatively, a few mm^2^ of blood-spotted filter paper) was added to 50 μL of lysis solution (200 mM KOH, 20 mM Na2EDTA, 0.25% Triton X-100); in fresh blood, complete lysis occurs within a few seconds, and it results in sudden increase of the solution viscosity, due to free DNA. However, further incubation at ambient temperature for 1–2 min after lysis improves DNA and protein denaturation and solubilization (some blood proteins, such as proteases, are known to inhibit DNA amplification), as well as the dissociation of DNA/proteins complexes.

Dry blood stains absorbed onto filter paper (air-dried and stored up to several months at room temperature) were incubated in lysis buffer at 70–80 °C for 10 min or until the blood appeared dissolved (alternatively, incubation can be carried out overnight at room temperature); feathers (one per sample) require harsher conditions and were incubated at 98 °C for 5 min or, alternatively, at 80 °C for 15 min, followed by 1 min at 98 °C.

**Figure 1:**
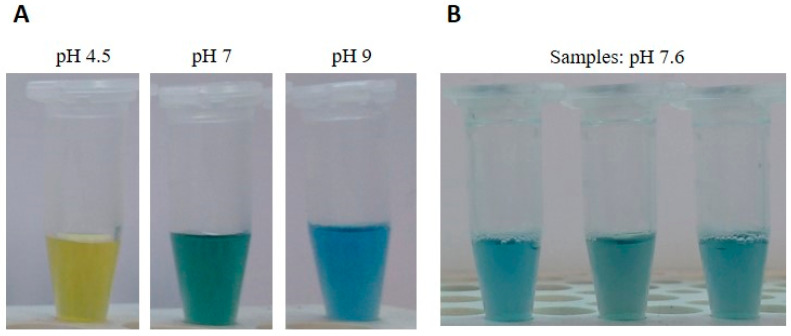
DNA extraction from blood samples. (**A**) Samples with different pH values, containing the bromothymol blue pH indicator (BTB). DNA samples appear yellow, green, or blue at acidic, neutral, or basic pH, respectively. (**B**) Color of solution at correct pH value (7.6).

After a brief, thorough mixing, six volumes of a BTB neutralization solution (50 mM Tris-HCl, 0.0025% bromothymol blue) were added and the samples were briefly mixed. This makes the use of micropipettes not strictly necessary, and both solutions can be measured as drops (six drops of neutralization solution per drop of lysis solution). The pH indicator BTB was previously tested for its compatibility with amplification reactions and was included to check samples for proper pH values, compatible with downstream applications. In fact, the dye turns from yellow to blue/green around pH values of 7.6 (Figure 1A), the correct pH is easily monitored by the color of the solution (Figure 1B) and the resulting buffer system ensures suitable pH values even if slightly different volumes are added.

### 2.2. Sex Determination by Standard PCR and DNA Sequencing

Sexing by standard PCR was carried out using the primers pair CHD2550F/CHD2718R [1]. The reaction mix consisted of 500 nM of each primer, 200 μM dNTPs, 1× Reaction Buffer, 0.2 μL of Phire Hot Start II DNA Polymerase (Thermo Scientific, Waltham, MA, USA), 1 μL of DNA from crude neutralized lysates, in a final volume of 25 μL.

Cycling conditions were as follows: 98 °C for 20 s, followed by 5 cycles of 98 °C for 7 s, 58 °C for 15 s and 72 °C for 20 s, and 35 cycles of 98 °C for 7 s, 50 °C for 10 s and 72 °C for 15 s, with a final extension step of 2 min at 72 °C. Reactions were performed in an iCycler (Bio-Rad) thermal cycler.

Amplicons for sequencing of the genomic regions of interest (Chromo-Helicase-DNA binding genes CHD-Z and CHD-W), whose nucleotidic sequences (needed for the design of specific primers) were not available in databases, were produced from one male and one female of each considered species by PCR, using the primers pair CHD2550_M13F/CHD2718R [1]. The CHD2550_M13F primer is a modified CHD2550F, bearing the universal M13F tag at the 5′ end, which allows for subsequent sequencing using the M13 universal primer.

Amplicons were excised and gel purified, using Illustra GFX PCR and Gel purification DNA Kit (GE Healthcare), and sequenced by the Sanger method by Macrogen Europe (The Netherlands).

Sequences have been submitted to GenBank and are publicly available with the following accession numbers: MF662597, MF662599, MF662601, MF662598, MF662600, MF662602.

Sexing with the newly designed primers for analysis onto 2% agarose gel was performed using the Proc-Fw/Proc-M-Rv/Proc-F204Rv primers mix (Proc-F343Rv can replace F204Rv) (Table 1), the PCR mix as above and the following cycling conditions: 98 °C for 20 s, followed by 35 cycles of 98 °C for 6 s, 60 °C for 10 s, and 72 °C for 10 s, with a final extension step of 1 min at 72 °C.

### 2.3. PCR Primers Design

**Figure 2:**
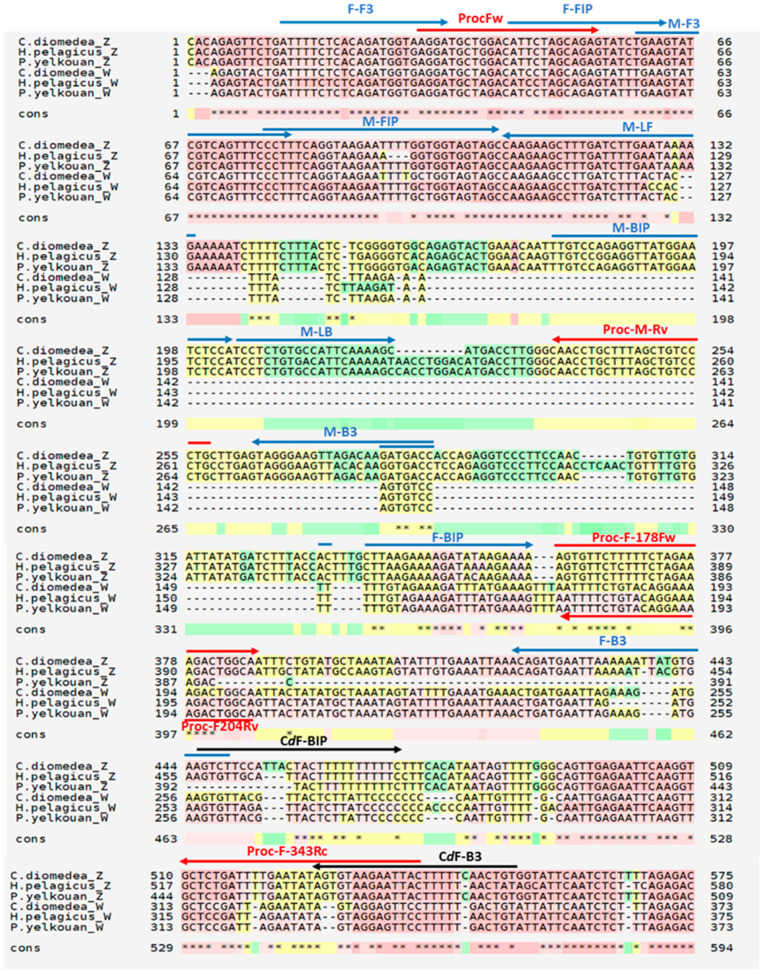
Multiple sequence alignment of CHD-Z and CHD-W of selected Procellariiformes using M-Coffe. Primer sets for PCR and LAMP reported in Table 1 are highlighted as red and blue arrows, respectively. Black arrows indicate *C. diomedea* female-specific LAMP primers.

PCR primers (Proc-Fw, Proc-M-Rv, Proc-F178Fw, Proc-F343Rv (Table 1, Figure 2)), were designed based on the aforementioned sequences of CHD-Z and CHD-W of the three Mediterranean Procellariiformes (*C. diomedea, P. yelkouan, H. pelagicus melitensis*, respectively), to target conserved, sex-specific sequences, after sequences alignment by ClustalOmega (http://www.ebi.ac.uk/Tools/msa/clustalo/ (accessed on 25 July 2020)) and M-Coffe (https://tcoffee.crg.eu/ (accessed on 25 July 2020)).

Primers were edited manually, checking their features by Oligo Analyzer v.1.0.2 Software (www.uku.fi/~kuulasma/OligoSoftware (accessed on 25 July 2020)), in order to improve their features. Noteworthy, it is crucial that primers to be used for this technique have optimal features (negligible self-annealing and primer-primer annealing, melting temperature (Tm) above 60 °C (optimal between 64–68 °C) and give amplicons not shorter than 150 bp (longer the amplicon, higher the fluorescence). Furthermore, primers were challenged, by a bioinformatic approach, with the other Procellariiformes for which the sequences corresponding to both sexes (CHD-Z and CHD-W) were available in GenBank, namely *Fulmarus glacialis* (GenBank Accession numbers: AY178142.1, KC788935.1) and *Oceanodroma leucorhoa* (GenBank Accession numbers: AY178147.1, AY178123.1).

### 2.4. LAMP Primers Design

Two LAMP primer sets, ProcZ and ProcW (specific for male and female chromosomes, respectively), targeting Mediterranean Procellariiformes (*C. diomedea, P. yelkouan, H. pelagicus melitensis*) were designed based on the aforementioned sequences of Chromo-Helicase-DNA binding CHD-Z and CHD-W gene of the three species, respectively, using the Primer Explorer V4 software (Eiken Chemical Co., Ltd., Tokyo, Japan; http://primerexplorer.jp/e/ (accessed on 15 May 2019)) (Table 1, Figure 2). Primers were further edited manually, in order to improve some sequence features. The two ProcZ and ProcW primers sets were selected with optimal features which allowed employing them at the same temperature with no risk of non-specific cross-amplification. Forward primers (F3, FIP) were designed on poorly polymorphic sequences, conserved in CHD-Z and CHD-W; backward primers target highly specific regions, found uniquely either in CHD-Z or CHD-W. The ProcZ mix also contains a couple of loop primers, which speeds up the reaction and improves the sensitivity in control reaction; unfortunately, despite several attempts, we could not find suitable loop primer pairs with any software settings employed during CHD-W sequence analysis. Moreover, we designed a further, female-specific, Scopoli’s Shearwater-targeted LAMP backward primers set (namely CdW, including CdF-B3 and CdF-BIP) with improved features that, combined with the Procellariiformes-, female-specific forward primers (namely, F-F3 and F-FIP), allowed for more efficient and faster reactions in this species (Table 1, Figure 2).

### 2.5. High-Performance PCR

Sexing with the newly designed primers for in-tube detection was performed using two primers sets (Table 1): Proc-Fw/Proc-M (M set) for the CHD-Z-specific reaction and Proc-F178Fw/Proc-F343Rv (F set) for the female-specific reaction. For both reactions, the PCR mix was prepared as described above, except for the addition of 4% dimethyl sulfoxide (DMSO), the primers concentration (increased to 750 nM), and the enzyme amount following the manufactures’ directions. Reactions were carried out with the following cycling conditions: 98 °C for 20 s, followed by 25 cycles of 98 °C for 5 s, 65 °C for 15 s, and 72 °C for 5 s; a further 15 cycles were performed with an annealing temperature of 60 °C, followed by a final extension step of 1 min at 72 °C. Five microliters of 1:100 water dilution of GelRed (Biotium Inc., Fremont, CA, USA) were added to each reaction and mixed thoroughly before reading under UV/Blue light. SYBR-Green I, which offers the advantage of being excited by blue light, can successfully replace GelRed. Good results were achieved using different cycling programs and template DNA amounts, as well (see Table S1 and Figure S1).

The method was first developed to sex the Scopoli’s Shearwater, then validated and tested in the other two Mediterranean Procellariiformes (which belong to two different Families, namely Procellariidae and Hydrobatidae), using DNA from blood as a template.

PCR primers were firstly tested using known-sex samples, then on new specimens.

The PCR yield was calculated by DNA quantification after gel electrophoresis of serial dilutions of PCR products in presence of GeneRuler DNA Ladder Mix (Thermo Scientific) as quantity standard.

**Figure 3:**
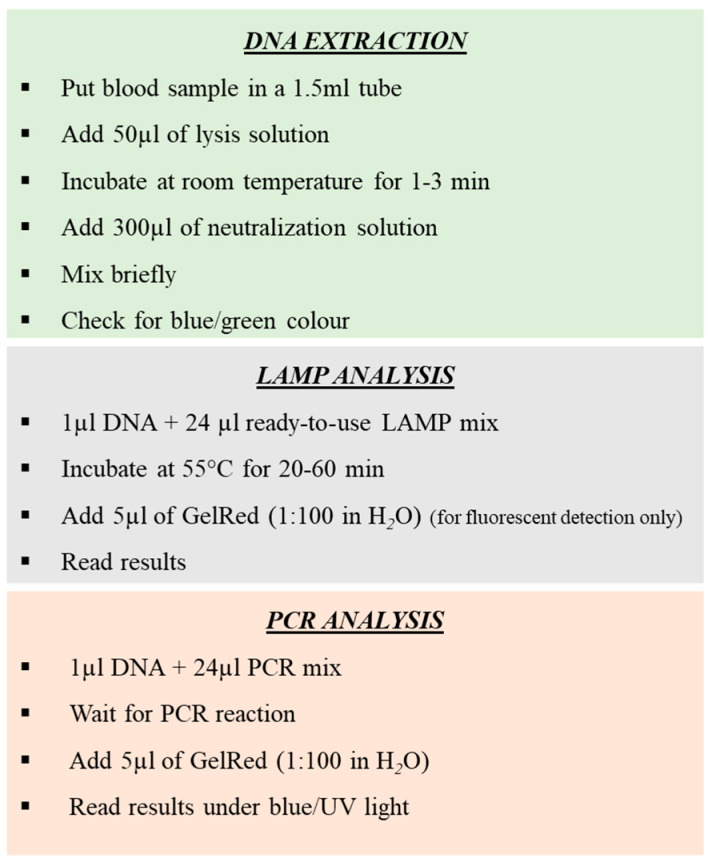
Step of amplification procedures for rapid sex determination of *Procellariiformes*, by loop-mediated isothermal amplification (LAMP) and high-performance PCR.

A step-by-step description of the entire procedure is summarized in Figure 3.

### 2.6. Sex Determination by LAMP

Two sets of primers, ProcZ and ProcW (Table 1), were employed. The correct amplification reaction with ProcW occurs specifically in females, as it targets sequences present only in CHD-W, whereas amplification with ProcZ has to be considered as a positive control, because it is expected to occur in both males (ZZ) and females (ZW), thus excluding any reaction failure due to lack of proper template and/or to the presence of inhibitors. Primers were first tested on known-sex samples of *C. diomedea* (10 males and 10 females), in order to verify their specificity. Results were verified, a posteriori, by PCR. The ready-to-use mix consisted of 1× enzyme buffer, dNTPs 1.4 mM, MgSO_4_ 8 mM (final total concentration), 1.6 µM each FIP/BIP, 0.2 µM each F3/B3, 0.4 µM each Loop F/B, eight units of Bst 2 Warm-Start DNA polymerase (New England Biolabs), 2% sucrose, 700 µM Eriochrome Black T (EBT), and 5% DMSO (optionally). The use of molecular biology-grade reagents (nucleases-free) is strongly recommended. LAMP reaction mixes to be used for in-door sexing (in laboratory) can be stored frozen omitting the addition of sucrose and enzyme. For those cases in which the mix has to be kept at room temperature only, the strategy described in Hamburger et al. (2013) [20] and Centeno-Cuadros et al. (2017) [11] can be employed, replacing DMSO with betaine. LAMP reactions were incubated for 20–60 min at 55 °C, with *C. diomedea* primers set; alternatively, an initial 5–10 min step at 65 °C can be introduced, which results in improved sensitivity [21]. Successful amplification was visually detected by color change, which could be checked also while reaction is proceeding, without the need of further reagents additions and samples manipulation after the reaction, whereas previously reported procedures include the addition of the dye to each sample at the end of the reaction [11]. In fact, EBT is a metallochromic indicator whose color changes depending on the dye/cation complexes formation; in particular, in the LAMP reaction it turns from red (Mg-EBT) to blue (free-EBT) as free Mg^++^ ions concentration drops, due to the formation of poorly soluble magnesium pyrophosphate associated to dNTPs hydrolysis during DNA synthesis. Alternatively, LAMP results can be read by fluorescence. In this case, each reaction tube can be added 5 μL of 1:100 water dilution of GelRed (Biotium Inc., Fremont, CA, USA), similarly to previous reports. After a brief mixing, the reaction turbidity increases and the color undergoes a slight change in the positive reactions; however, sharpest results are visualized under UV light, where positive samples exhibit a strong bright red-orange fluorescence. During preliminary tests, in order to ensure the color change or fluorescence were due actually to specific DNA amplification, LAMP-amplified products, as well as negative controls, were analyzed by electrophoresis on 3% agarose gel.

## 3. Results

### 3.1. Optimization of “High-Performance PCR” Reaction

**Figure 4:**
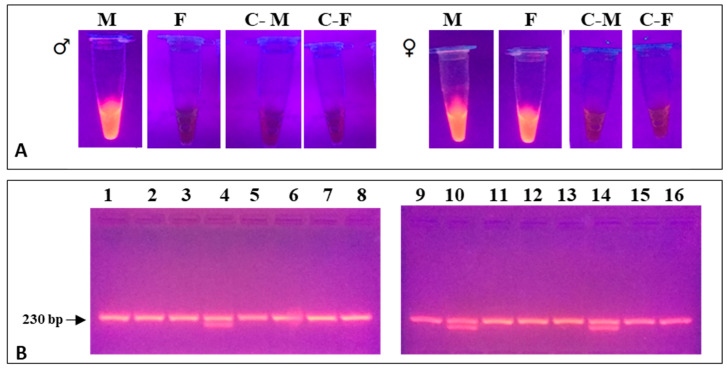
DNA (**A**) Fluorimetric detection of PCR products using the set of primers M and F under UV light using GelRed. Blue light gives similar results (**B**) agarose gel electrophoresis of PCR products obtained using the newly designed primers (Proc-Fw/Proc-M-Rv/Proc-F204Rv) for single-tube reaction on randomly selected samples of *C. diomedea* (Table 1). Line 1, 2, 3, 5, 6, 7, 8, 9, 11, 12, 13, 15, 16: male samples blood; Line 4, 10, 14: female samples blood. C- are the negative controls.

The method developed for PCR sexing results in na very short reaction time and DNA yields that are high enough (>100 ng/µL) to achieve fluorescence levels detectable by naked eye under proper light. The method, using DNA from blood, allowed for a fast and reliable identification of males and females (Figure 4A), which could be confirmed in 200 samples by also the standard universal primers pair CHD2550F/CHD2718R [1]. Thereafter, the method was employed on more than 1200 *C. diomedea* samples, among which only 3 (less than 0.3%) failed to amplify; we have had no evidence of sex misidentifications, so far. Moreover, some of the new primers could be combined in a single reaction for faster sexing by standard PCR followed by agarose gel electrophoresis (Figure 4B). The length of the amplicons, 227 and 164 bp for males and females, respectively, much lesser than that obtained using the aforementioned primers pair (exceeding 700 and 450 bp, respectively), allows the new primers to be suitable also for PCR analyses of samples containing partially degraded DNA. It was tested using feather samples (collected more than 10 years earlier; N = 130), all of which could be successfully sexed, whereas universal primers (CHD2550/CHD2718) had failed. The PCR method produced the same results in the other two Mediterranean Procellariiformes, as expected. The analytical method proved to be robust and reliable in a relatively wide range of experimental conditions (i.e., temperatures, cycling programs, DNA concentrations; see Figure S1); the Materials and Methods section reports those that, in our hands, performed better.

### 3.2. Optimization of LAMP Reactions in Pelagic Birds

**Figure 5:**
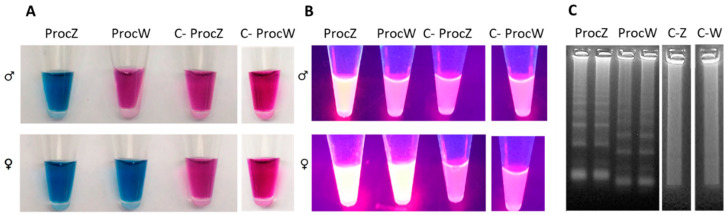
Visual detection of LAMP products using the set of primers ProcZ-ProcW on *C. diomedea*. (**A**) Detection using EBT: the color of the reaction with daylight was blue when LAMP reaction was positive and remained red when there was no amplification and in the negative control (C-). ProcZ mix reaction amplified both males and female samples; ProcW mix was specific for female samples. (**B**) Fluorimetric detection using GelRed. The presence of fluorescence is observed in ProcZ mix reaction, specific for males and female samples; no fluorescence signal is observed in male samples amplified with ProcW mix and in the negative control. (**C**) PCR products were observed in agarose gel by electrophoresis analysis. C-Z; C-W) negative control ProcZ and ProcW mix, respectively.

The method allowed the full discrimination of males and females in all the tested species and was proven to be reliable in a wide range of temperatures (50–60 °C) and short times (20–60 min), owing to the high specificity and efficiency of primers. It was noteworthy that females of all analyzed species were correctly identified, as confirmed by PCR analyses. Although 20–30 min was usually enough for the correct sex assessment using the lowest reaction temperatures, increasing the reaction time to 60 min resulted in higher tube brightness, using fluorescence. Sex was determined by simply reading the color array in analysis tubes. In male samples, the blue color is observed in one tube only, as the second (containing the ProcW mix) fails to amplify and remains red, similar to the negative control. In contrast, amplification occurs in both tubes if analyzing females (Figure 5A). Similarly, fluorescence is observed in one or two tubes for males and females, respectively, if using GelRed for fluorimetric detection, instead of EBT (Figure 5B). PCR amplification with universal primers confirmed sex determination for all samples (N = 80, during validation) analyzed by LAMP (Figure 5). The method produced the same results in the Mediterranean Storm Petrel and the Yelkouan Shearwater, as expected. Neither sex misidentifications nor amplification failures have been recorded. Thereafter, a further primer set, specific for Scopoli’s shearwater was designed (Figure 2). We exploited some unique (not shared) CHD-W specific sequences located beyond the 3′ border of conserved regions across the three analyzed taxa, which allowed the design of more stable primers for faster and more robust analysis.

## 4. Discussion

We have developed a simple and robust PCR method that allows in-tube visual reading of results, named high-performance PCR (hp-PCR). We applied it to the detection of CHD1 alleles for molecular sexing of pelagic birds. Moreover, we set up an improved test based on loop-mediated isothermal amplification (LAMP) aiming at overcoming some limits of the existing one for sexing of Procellariiformes. The hp-PCR method shows several advantages over standard PCR-based ones, allowing to sex birds in less than one hour, combining a fast and efficient optimized DNA extraction, with a high-performance DNA amplification reaction. This allows for immediate detection of results by the unaided eye, without the use of DNA separation techniques, for an overall improved workflow.

In particular, we first developed a method of DNA extraction from feather and blood samples, specifically formulated to reduce times and to optimize the yield and quality of the DNA template for subsequent amplification reactions.

Previously, other alkaline treatments have been successfully employed to achieve either cell lysis [21] or PCR inhibitors inactivation [19], which makes such an approach the potential lysis method of choice when many samples have to be analyzed for routine analyses. Indeed, such a system allows fast cell lysis, protein denaturation, RNA hydrolysis and removal of some PCR inhibitors, allowing direct amplification from various tissues and animal matrices [17]. Centeno-Cuadros et al. [11] reported the development of a protocol for DNA extraction from blood samples of raptor species based on NaOH, and leading to NaCl generation after neutralization, which may exert a concentration-dependent inhibitory effect on DNA polymerases [22]. We instead, optimize the DNA extraction protocol from feathers and blood of birds to decrease the inhibitors per reaction and to ensure successful PCR reactions. Indeed, KCl (which forms in the herein reported system) is well-known to stimulate Taq DNA polymerase activity over a broad concentration range, so that it is commonly included as a component of most PCR buffers. Moreover, Triton X-100, a comparatively mild non-ionic detergent often present in several amplification buffers, was included so that its concentration in the lysis solution is enough to enhance tissue lysis and drops down to that used as reaction additive in next steps. Moreover, the final EDTA concentration in reaction mixture proven to be devoid of any inhibitory effect on both PCR and LAMP.

Thereafter, we set up the PCR-based approach that relies on high-performance PCR, where short PCR amplification time and high DNA yields could be achieved by combining the use of highly efficient and specific primers, optimized reaction conditions and engineered, performant, highly sensitive and processive thermostable DNA polymerases for improved PCR efficiency in the shortest reaction time. The very high DNA yield allows for direct visualization by blue/UV light after staining of the reaction mix with a DNA-binding fluorochrome, making the immediate molecular sexing with minimal equipment possible, even using PCR instead of LAMP.

We sequenced the CHD1-W and CHD1-Z regions of selected species and designed two primer sets targeting specifically them, which allowed us to amplify separately the two alleles by hp-PCR. In the amplification reaction, CHD-Z and CHD-W specific regions (that we found to be conserved among Procellariforms) are amplified in separate reaction tubes using specifically designed primers and optimized conditions. Besides, the use of very short target regions allowed for successful analyses even on samples in which the extent of DNA degradation results in failure of PCR using the standard primers pair CHD2550F/CHD2718R. The use of allele-specific primers (in particular, the female-specific one) is expected to further improve the sensitivity and robustness of the analysis. Moreover, based on bioinformatics analyses carried out on the few available sequences from other Procellariiformes (namely, *Fulmarus glacialis* and *Oceanodroma leucorhoa*), the method is expected to be suitable for sexing of other species and, presumably, of most Procellariiformes. Bioinformatic analyses extended to the class of birds, showed sequence conservation in several other species across various orders, which expand the suitability of the PCR protocol to a much wider range of species. In particular, we could identify further species targeted by the reported primers including—but not limited to—Ciconiiformes, such as *Ciconia boyciana* and *C. Ciconia*; Gruiformes, such as *Grus grus, G. japonensis*, and *Balearica pavonine*; Accipitriformes, such as *Accipiter gentilis* and *Aquila chrysaetos*; and Falconiformes, such as *Falco peregrinus*. Unfortunately, based on publicly available sequences, the broad sequence variation observed across different species makes it difficult to conceive “true universal” primers suitable for such a technique, whereas it seems much more suitable the design of primers targeting selected (groups of) species. In this perspective, we have provided information and criteria to be followed in the design of further primer sets suitable for high-performance PCR. Besides targeting other taxa, further conceivable improvements might include the selection of longer target sequences to achieve higher sensitivity due to stronger fluorescence (though with negligible increases in the reaction time, if any). Moreover, the use of two primer pairs, each specific for the shared (CHD-Z) or the female-specific (CHD-W) allele, makes suitable target regions conserved across different taxa relatively easier to find. This helps overcome a common limit of the LAMP analysis, in which at least six target sequences are required.

Overall, the direct visualization of results in hp-PCR allowed for immediate reading of results without gel electrophoresis, which makes this technique particularly suitable for the analysis of large batches of samples. In this setting, avoiding the gel electrophoresis step may greatly speed up the overall workflow, making it potentially reliable even outdoor.

This is, to our knowledge, the first report of such an approach, making the broader use of PCR possible as a versatile, reliable, and cost-effective technique, suitable not only for rapid molecular sexing, but worthy to be tested for many other applications in which the rapid detection of a specific sequence is required. Noteworthy, although we report a protocol tailored (firstly due to the species-specific primers) for Procellariiformes, we point out that the method itself can be implemented and extended to several other species, as discussed.

Molecular sexing of birds using the LAMP technology shows advantages over standard PCR-based approaches, including the possibility to perform analyses in the field and to get rapid results with a few operational steps. The LAMP primer set developed here allows to overcome the limits (i.e., the failure of amplification in control reaction) reported for sexing of Procellariiformes using the existing protocol [7], which remains the only technique proposed for outdoor molecular sexing so far [7,12]. The issues and limits in the design of the primers arising from the not complete conservation of target sequences across species, together with the need to meet the primers’ features requirements, highlights the concept that highly efficient, universal primers set are difficult to be conceived, which hampers the broader use of primers sets in across taxa and genera. Thus, although the extension of the method to higher taxonomic levels would be desirable, the LAMP approach should be primarily intended as specific for selected taxa.

In conclusion, the analytical methods proved to be robust and reliable, allowing for rapid bird sexing. Nevertheless, as the fluorimetric “in-tube” detection in hp-PCR relies on the actually optimal PCR performances and DNA yield, we strongly recommend a careful setup of the reaction conditions, as they may depend—as it is well-known—on the specific thermal cycler employed and reagents. Instead, a much lesser sensitivity to analytical conditions is expected—and was observed—for single-tube PCR reactions to be analyzed by gel electrophoresis, where even suboptimal conditions do not affect the interpretation of the results and, in the worst case, result in slightly less bright bands.

Overall, the approaches described herein provide wildlife managers, veterinarians, researchers, as well as breeders, robust, reliable, and tailored tools for fast sexing of a broader number of species. Moreover, the high-performance PCR shall be implemented to be extended to any applications requiring the fast and efficient detection of specific targets, including—but not limited to—species identification, diagnostic and/or confirmation tests, etc.

## Figures and Tables

**Table 1:** Primer sets used in the study to amplify CHD-Z and CHD-W of three Mediterranean Procellariforms (*Calonectris diomedea, Puffinus yelkouan, Hydrobates pelagicus*) by PCR (M and F primer set) and LAMP (ProcZ and ProcW primer set) methods. M and F set were used for male and female-specific reaction, respectively. ProcZ occurs in both males and females, ProcW occurs only in females. Nucleotide positions are based on *C. diomedea* CHD-Z and CHD-W sequences.

Method	Primers Set	Primer	Sequence (5′-3′)	Nucleotide Position (5′-3′)	References
PCR	M	ProcFw	AGGATGCTRGACATYCTAGCAGAG	30–54	This study
		Proc-M-Rv	CAGGGACAGCTAAAGCAGGTTG	237–257	
	F	Proc-F178Fw	ATTTTCTGTACAGGAAAAGACTGGCA	177–202	
		Proc-F343Rv	GGAACTCCTACTATATTCTAATCGGAGC	313–340	Fridolfsson and Ellegren 1999 [1]
		Proc-F204Rv	TGCCAGTCTTTTCCTGTACAGAAAAT	177–202	
	Universal	CHD2550	GTTACTGATTCGTCCACGAGA		
		CHD2718	ATTGAAATGATCCAGTGCTTG		
		M13F-Tag	TTGTAAAACGACGGCCAGT		
LAMP	ProcZ	M-F3	TGAAGTATCGTCAGTTTCCCTT	59–80	This study
		M-B3	GGTCATCTTGTCTAACTTCCCTAC	263–284	
		M-FIP	CCACCCCGAGAGTAAAGAAAAGATTTTTTTCAGGTAAGAATTTTGGTGGTAGTAGC	78–107	
		M-BIP	TTGTCCAGAGGTTATGGAATCTCCATTTTAGCAGGTTGCCCAAGGTC	178–203	
		M-LF	CTTTTATTCAAGATCAAAGCTTCTTG	108–133	
		M-LB	TCCTCTGTGCCATTCAAAAGC	204–224	
	ProcW	F-F3	ATTTTCTCTCAGATGGTGAGGA	9–31	
		F-B3	ACACTTCATCTTTCTAATTCATCAGT	235–261	
		F-FIP	GCTACTACCAGCAAAATTCTTACCTGTTTTCATCCTAGCAGAGTATTTGAAGTATCG	38–65	
		F-BIP	GTGTCCTTTTTGTAGAAAGATTTATGAAAGTTTTAGCATATAGTAATTGCCAGTCTTTTCC	143–173	
	CdW	*Cd*F-B3	ACAGTCAAAAAGGAACTCCTACT	329–351	This study
		*Cd*F-BIP	AGTGTTACGTTACTCTTATTCCCCCTTTTTCTAATCGGAGCAACTTGAA	257–276	

## Data Availability

Not applicable.

## Supplementary Materials

The following supporting information can be downloaded at: https://www.mdpi.com/article/10.3390/biology12020300/s1, Figure S1: Fluorimetric detection of PCR products obtained using alternative hp-PCR conditions (T1–T2) with different annealing temperatures and cycling programs listed in Table S1, and results obtained using a 1:20 dilution of template DNA for amplification with the standard (T0) hp-PCR program (T0 DNA 1:20). Table S1: PCR conditions used in this study. Conditions that differ from T0 are shown in bold italic style.
