# Supplementary material for: High-Performance PCR for Alleles Discrimination of Chromo-Helicase-DNA Binding Protein (CHD1) Gene in Bird Sexing
Source: Biology (Basel). 2023 Feb 14;12(2):300. doi: 10.3390/biology12020300 (PMC9953129; doi:10.3390/biology12020300)
Supplement: Supplementary file 1 [file biology-12-00300-s001.zip › biology-2106259-supplementary.pdf]

Supplementary

Table S1. PCR conditions used in this study. Conditions that differ from T0 are shown in bold italic style.

**PCR condition Trial 0 (T0)**		
98 °C (Preheating)	20 s	
98 °C (Denaturation)	5 s	25 cycles
65 °C (Annealing)	15 s	
72 °C (Extension)	5 s	
98 °C (Denaturation)	5 s	
60 °C (Annealing)	15 s	15 cycles
72 °C (Extension)	5 s	
72 °C (Extension)	1 min	
**PCR condition Trial 1 (T1)**		
98 °C (Preheating)	20 s	
98 °C (Denaturation)	5 s	25 cycles
68°C (Annealing)	15 s	
72 °C (Extension)	5 s	
98 °C (Denaturation)	5 s	
58 °C ± 1 (Annealing)	15 s	15 cycles
72 °C (Extension)	5 s	
72 °C (Extension)	1 min	
**PCR condition Trial 2 (T2)**		
98 °C (Preheating)	20 s	
98 °C (Denaturation)	5 s	***30 cycles***
**62 °C** (Annealing)	15 s	
72 °C (Extension)	5 s	
98 °C (Denaturation)	5 s	
60 °C (Annealing)	15 s	***10 cycles***
72 °C (Extension)	5 s	
72 °C (Extension)	1 min	

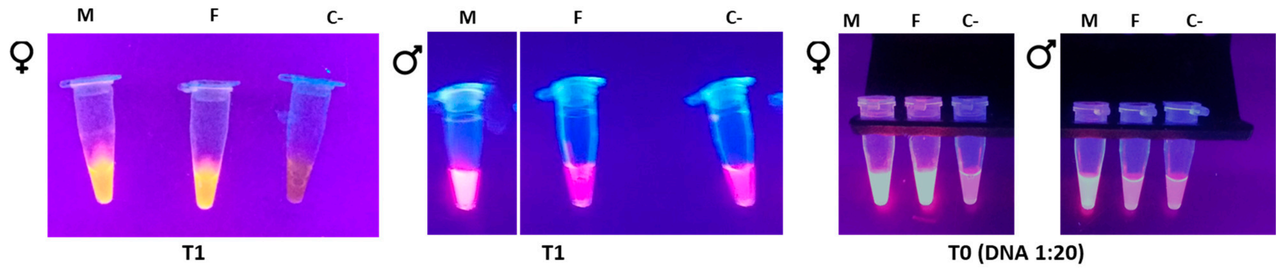

Figure S1. Fluorimetric detection of PCR products obtained using alternative hp-PCR conditions (T1–T2) with different annealing temperatures and cycling programs listed in Table S1, and results obtained using a 1:20 dilution of template DNA for amplification with the standard (T0) hp-PCR program (T0 DNA 1:20).
